# Lactoferrin in Human Milk of Prolonged Lactation

**DOI:** 10.3390/nu11102350

**Published:** 2019-10-02

**Authors:** Matylda Czosnykowska-Łukacka, Magdalena Orczyk-Pawiłowicz, Barbara Broers, Barbara Królak-Olejnik

**Affiliations:** 1 Neonatology Department, Wroclaw Medical University, Borowska 213, 50-556 Wroclaw, Poland; barsamut@gmail.com (B.B.); barbara.krolak-olejnik@umed.wroc.pl (B.K.-O.); 2 Department of Chemistry and Immunochemistry, Wroclaw Medical University, M. Skłodowskiej-Curie 48/50, 50-369 Wrocław, Poland

**Keywords:** breastfeeding, lactoferrin, prolonged lactation, child nutrition

## Abstract

Among the immunologically important bioactive factors present in human milk, lactoferrin (Lf) has emerged as a key player with wide-ranging features that directly and indirectly protect the neonate against infection caused by a variety of pathogens. The concentration of Lf in human milk is lactation-stage related; colostrum contains more than 5 g/L, which then significantly decreases to 2–3 g/L in mature milk. The milk of mothers who are breastfeeding for more than one year is of a standard value, containing macronutrients in a composition similar to that of human milk at later stages. The aim of this study was to evaluate lactoferrin concentration in prolonged lactation from the first to the 48th month postpartum. Lactating women (*n* = 120) up to 48 months postpartum were recruited to the study. The mean value of lactoferrin concentration was the lowest in the group of 1–12 months of lactation (3.39 ± 1.43 g/L), significantly increasing in the 13–18 months group (5.55 ± 4.00 g/L; *p* < 0.006), and remaining at a comparable level in the groups of 19–24 month and over 24 months (5.02 ± 2.97 and 4.90 ± 3.18 g/L, respectively). The concentration of lactoferrin in mother’s milk also showed a positive correlation with protein concentration over lactation from the first to the 48th month (*r* = 0.3374; *p* = 0.0002). Our results demonstrate the high immunology potential of human milk during prolonged lactation and that Lf concentration is close to the Lf concentration in colostrum. Evidence of stable or rising immunoprotein levels during prolonged lactation provides an argument for foregoing weaning; however, breastfeeding must be combined with solid foods meet the new requirements of a rapidly growing six-month or older baby.

## 1. Introduction

Human milk (HM) serves as the gold standard in newborn and infant nutrition. Apart from the main nutrients, HM contains many bioactive proteins, growth factors, cells, and other constituents, which are crucial in the modulation of the development of a competent immune system to protect the term and preterm newborn against pathogens [1]. Among the immunologically important bioactive factors present in human milk, lactoferrin has emerged as a key player with wide-ranging features that directly and indirectly protect the neonate against infection caused by bacteria and other microorganisms.

Lactoferrin (or lactotransferrin, Lf) is an approximately 78 kDa glycoprotein from the transferrin family of proteins. Lf was first identified by Sørensen and Sørensen in 1939 in bovine milk as a red protein in whey [2], and later isolated in 1960 from both human and bovine milk [3,4,5]. Lf is expressed and secreted by epithelial cells in the main physiological secretions [6,7,8]. Lf plays a key role in specialized systems to bind iron, affecting cellular proliferation and differentiation, dependent on the degree of iron saturation [9,10]. Clinical trials have demonstrated roles for Lf in the prevention of diarrhea [11,12], neonatal sepsis, and necrotizing enterocolitis in preterm infants [13,14,15]. Lf may significantly impact development as well as the well-being of infants and later outcomes, which are directly related to breastfeeding. Maternal factors may affect the concentration of Lf in human milk, but affect infant factors as well. Lf concentrations are consistently highest in colostrum, then decrease gradually. However, the specific factors that are related to the Lf concentration in breast milk are unknown. Factors such as geographical location, ethnic origin, genetic polymorphisms, socio-economic status, nutritional status, as well as infants infections, type of delivery, and prematurity have been considered [16,17,18].

In human milk, Lf is the most abundant protein in the whey fraction [7]. The concentration of Lf in human milk is lactation-stage related: colostrum contains more than 5 g/L, which then significantly decreases to 2–3 g/L in mature milk. Multiple studies have evaluated Lf concentrations in human colostrum and mature milk and in term and preterm milk [7,19,20].

Lf elicits antibacterial effects against bacteria; however, Lf has protective activity due to the high affinity to iron. The iron-free form of Lf (apo-Lf) is a cause of iron deficiency in microorganisms as iron is required for life and growth rate [21]. Research has shown that the direct interaction between Lf and bacteria results in a bactericidal effect [22]. Lf associates with the lipoprotein of bacterial cells and forms receptor complexes. The binding of Lf inhibits the iron intake of bacteria or removes the protective effect of the membrane. Lf causes the release of lipopolysaccharides from the cell’s wall, increases permeability of the membrane, and finally kills the Gram-negative bacteria. Lf binds the anionic molecules (e.g., lipoteichoic acid) on the cell surface of Gram-positive bacteria with greater effectiveness than lysozymes and antibiotics [23]. The antiviral effect of Lf is observed in the early phase of infection by inhibition of the growth of viruses. Lf and Lf-derived peptides can effectively act on a broad spectrum of fungal species, such as *Candida albicans, Candida krusei,* and *Aspergillus fumigatus* [24] by increasing membrane permeability, which leads to their death and additionally produces iron deprivation effects. Lf has a positively charged surface, which produces an anti-inflammatory effect. Lf interacts with proteoglycans on the surface of immune cells. This association can trigger signaling pathways that lead to physiological anti-inflammatory responses [25,26].

With reference to the research on the evolution of mammals, the protective function was the first in the ancestral mammary gland, which subsequently evolved to nourish the offspring. The protective components of milk remain highly conserved [27,28].

Breast milk after one year of lactation has nutritional value for children [29]. According to our previous results, the milk of mothers who breastfeed their children over one year or even over two years is standard value milk, and its macronutrient quantity composition is comparable mother’s milk up to the first year of lactation [29]. However, further detailed quantitative data on the concentration and variability of the most important bioactive and immunomodulatory human milk proteins are necessary. The aim of this study was to evaluate lactoferrin concentration, the main glycoprotein with anti-pathogen activity, in breast milk during prolonged lactation from 1–48 months of lactation and to identify if any correlation exists between lactoferrin and protein concentrations during a healthy mother’s lactation period. This is particularly important because Lf is an element providing innate immunity that is transferred to the breastfed infants and is crucial in the shaping and development of its immature immunological system.

## 2. Material and Methods

Mothers during lactation were recruited to the study from February 2017 to June 2018 using local groups for breastfeeding women on Facebook. We enrolled 120 participants in the study. The mother’s age, socioeconomic status, race, health status, concomitant medications, parity, the mode of delivery, and frequency of breastfeeding were recorded. Milk samples were collected at the Regional Human Milk Bank and University Hospital (Wroclaw, Poland) between 08:00 and 14:00. Providing an interval of a few hours provides greater uniformity of samples. Considering efficiency of milk expression, an electric breast pump was used (Medela Symphony, Baar, Switzerland). Each mother received a sterile set and cup for milk collection. Samples for analyses ware taken immediately after complete emptying of the breast (two aliquots of 2–3 mL each). One session of milk expression should not deprive the infant of their nutritionally required volume. Milk samples were divided into four groups according to months postpartum: the first group was up to 12 months (*n* = 24), second group from 13 to18 months (*n* = 33), third group from 19 to 24 months (*n* = 37) and last group beyond 24 months (*n* = 26). The milk samples after collection were immediately cooled and frozen in −20 °C. This study received ethical approval Nr KB–65/2018 from the University Ethics Committee. Informed and written consent was provided by all participants before sample collection.

### 2.1. Analysis of the Samples

Each sample was initially heated at 40 °C as recommended by the human milk analyzer producer and homogenized using a sonicator (Sonicator^®^, MIRIS, Uppsala, Sweden) at 1.5 s/mL, to separate the lipid phase and avoid protein aggregation. Each aliquot was homogenized differently immediately prior to measurement. Analyses were performed in triplicate. Breast milk macronutrient concentration was measured using a human milk analyzer (HMA) (MIRIS, Uppsala, Sweden) calibrated previously with human milk standards. The HMA is based on mid-infrared spectroscopy, enabling the assessment of fat, protein, lactose, energy, and total solids content. Protein is the protein content based on the total amount of nitrogen (N) in a sample. This means that non-protein nitrogen (NPN) compounds are also included in this value. “True protein” is corrected for this and represents only the content of actual protein, hence the denotation “true”. The Miris HMA (Uppsala, Sweden) measures both crude protein and true protein to avoid misunderstandings. The obtained results are expressed in g/L. A detailed description of the measurement method was described in previous study [29].

### 2.2. Skim Milk Sample Preparation

All collected samples of mother’s milk were immediately transferred to the dedicated plastic tubes WHAT for freezing and stored at −20 °C until analysis. To obtain the aqueous phase of milk (skim milk), all milk samples were centrifuged at 3500× *g* at 4 °C for 35 min. Next, the milk fat layer and cells were removed [30]. The skim milk samples were aliquoted and stored at −20 °C. All milk samples were handled by the same person. Before the determinations of lactoferrin concentration, the skim milk samples were thawed at room temperature for one hour.

### 2.3. Analysis of Lactoferrin in Skim Milk

The concentration of lactoferrin in skim milk samples was determined according to a modified procedure reported previously [31]. For testing, 100 µL of 5 × 10^3^, 10 × 10^3^, 25 × 10^3^, and 50 × 10^3^ fold diluted skim milk samples and parallel standard of lactoferrin derived from human milk in concentrations ranging from 0.8 to 50 ng per 100 µL (Sigma Aldrich, St. Louis, MO, USA) in Tris-HCl buffer with sodium chloride (pH = 7.5) were added to the individual microtiter plate wells (Nunc International, Naperville, IL, USA) and incubated for 2 h at 37 °C. The concentration of lactoferrin was analyzed using rabbit anti-human lactoferrin antibodies phosphatase-labeled (Jackson ImmunoResearch Europe Ltd., Ely, UK) in TRIS-HCl buffer (pH = 7.5) with 0.2% Tween 20 for 1 h at 37 °C and then detected by the reaction with phosphatase’s substrate, 4-nitrophenyl phosphate, disodium salt (SERVA, Heidelberg, Germany). The absorbance was measured after stopping the enzymatic reaction in a Stat Fax 2100 for Microplate Reader (Awareness Technology Inc., Palm City, FL, USA) using a 405 nm filter and 630 nm as the reference filter. The background absorbance for the ELISA test was low (below 0.04 AU) when Tris-buffered saline was added to the microtiter plate well instead of milk or lactoferrin standard samples. As the negative control, a human albumin (Sigma, St. Louis, MO, USA) was used. All skim milk samples were assayed at four different skim milk sample dilutions in duplicate. The intra-assay and inter-assay coefficients of variation were 4.8% and 5.6%, respectively.

### 2.4. Statistical Analysis

The statistical analysis was performed using the TIBCO STATISTICA 13.3 software package (StatSoft, Inc., Tulsa, OK, USA). The chi-square test was used for comparison the study population data. Due to the higher interindividual differences reported for many factors of human milk as well as the unequal sample size in the analyzed groups, nonparametric tests were used for analysis. Comparisons between analyzed groups were performed using the Kruskal–Wallis test. The obtained data are presented as the mean ± SD and the median with 25th–75th percentiles. To determine whether any relationships existed among the concentration of lactoferrin and analyzed factors, the correlations were evaluated according to Spearman’s coefficient. A *p*-value lower than 0.05 was considered significant. The charts were prepared using linear regression with a 95% confidence level.

## 3. Results

The key characteristics of the cohort are detailed in Table 1. All infants were exclusively breastfed up to six months.

### 3.1. Lactoferrin

**Figure 1:**
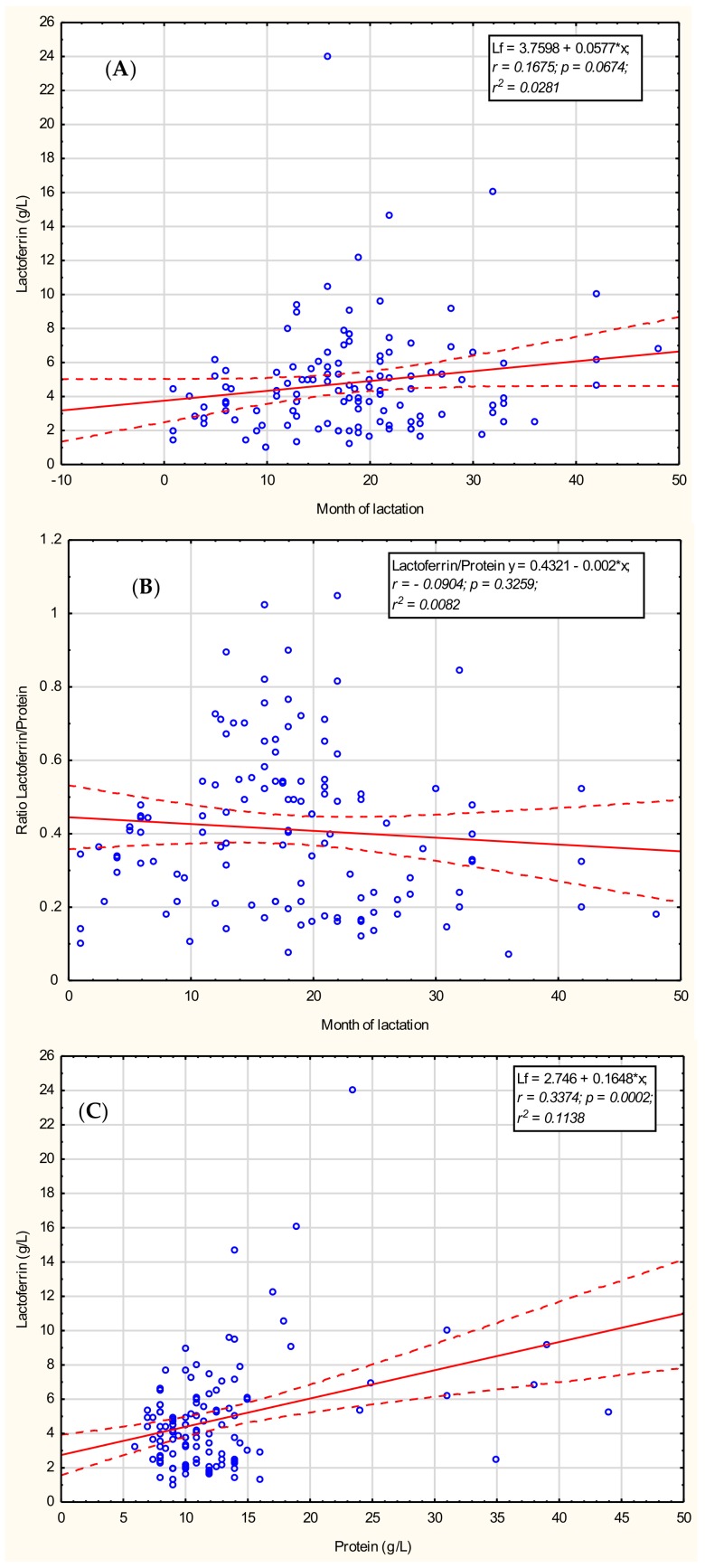
The correlation of (**A**) lactoferrin and (**B**) the ratio of lactoferrin to protein concentration with lactation progression from the 1st to the 48th month and (**C**) the correlation of lactoferrin and protein concentrations for all analyzed samples. A solid line indicates linear regression, and 95% confidence intervals are shown by dotted lines; blue hollow—individual samples; solid circle—2 or more individual samples with the same or very close values. *: multiplied

To determine if the concentration of lactoferrin in mother’s milk is connected with the month of prolonged lactation, the correlation was calculated; however, the obtained result showed no correlation (*r* = 0.1675; *p* = 0.0674) with prolonged lactation from the first to the 48th month, as shown in Figure 1A.

The mean value of lactoferrin concentration was the lowest value in the group of 12 months of lactation (3.39 ± 1.43 g/L), significantly increasing in the 13–18 months group (5.55 ± 4.00 g/L; *p* < 0.006) and remaining stable thereafter in the groups of 19–24 months and over 24 months (5.02 ± 2.97 and 4.90 ± 3.18 g/L, respectively) (Table 2).

### 3.2. Total Protein

The concentration of protein in mother’s milk was positively correlated with prolonged lactation during the analyzed period up to 48 months; however, the correlation coefficient value was weak (*r* = 0.25; *p* < 0.05).

The mean values of protein concentrations were comparable in the groups of 1–12 and 13–18 months of lactation (10.5 ± 2.3 and 10.4 ± 3.4 g/L, respectively). In subsequent analyzed lactation periods, i.e., 19–24 and over 24 months of lactation, protein concentrations significantly increased and reached 11.2 ± 2.7 g/L (*p* < 0.05) and 19.1 ± 10.7 g/L (*p* < 0.0001), respectively (Table 2).

### 3.3. Lactoferrin/Protein Ratio

To identify the existence of any relationship between the concentration of lactoferrin and the concentration of total protein, the correlation coefficient was calculated. Mother’s milk lactoferrin was positively correlated with protein concentration (*r* = 0.3374; *p* = 0.0002), as shown in Figure 1B, for the analyzed set of samples derived for 1–48 months of lactation. However, for both lactoferrin and protein concentrations, high interindividual differences among mothers were observed and this result may be characteristic for our particular data set only.

**Figure 2:**
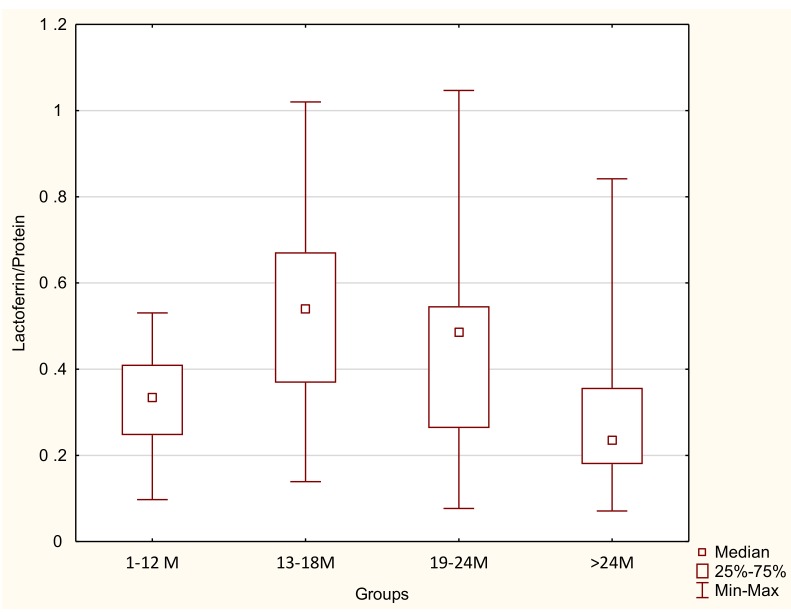
The coefficient of lactoferrin to the protein concentration in the four groups analyzed: Group1 up to 12 months (*n* = 24), group 2 from 13 to 18 months (*n* = 33), group 3 from 19 to 24 months (*n* = 37), and group 2 above 24 months (*n* = 26).

In contrast, the calculated coefficient of lactoferrin to the protein concentration (Lf/Protein) showed no correlation with lactation progression for 1–48 months (*r* = −0.0904; *p* = 0.3259) as shown in Figure 1C. The calculated coefficient of Lf/Protein in the group of 1-12 months of lactation reached 0.32 ± 0.12, significantly increasing in the 13–18-month group (0.49 ± 0.21, *p* < 0.002) and remaining at a comparable level in the 19–24-month group (0.43 ± 0.21), decreasing in the >24 months group to 0.29 ± 0.17 (*p* < 0.009; Table 2, Figure 2).

### 3.4. Lactoferrin Concentration in Relation to Macronutrients

The dependencies between lactoferrin concentration and macronutrients in mother’s milk were analyzed throughout the whole lactation period, 1–12, 13–18, 19–24, and >24 months of lactation. The values of the calculated coefficient, which are statistically significant (*p* < 0.05), are given in Table 3. In contrast with the lack of correlation between lactoferrin concentration and month of lactation for 1–48 months of lactation, a positive correlation was found for prolonged lactation, specifically above 24 months. The concentration of lactoferrin over 24 months is negatively correlated negatively with the concentration of carbohydrates (*r* = −0.50) and positively with the concentration of fat (*r* = 0.58), protein (*r* = 0.56), true protein (*r* = 0.58), dry mass (*r* = 0.65), and energy (*r* = 0.65). A statistically significant negative correlation was found between lactoferrin concentration and carbohydrates (*r* = −0.32) and positive correlations between lactoferrin and fat (*r* = 0.19), protein (*r* = 0.25), true protein (*r* = 0.24), dry mass (*r* = 0.18), and energy (*r* = 0.19) in the lactation from month 1 to 48. However, the correlations observed were much stronger in prolonged lactation above the month 24. In contrast, no correlations were found in the other analyzed periods of lactation, namely up to month 12, months 13–18, and 19–24.

### 3.5. Lactoferrin Concentration in Breast Milk in Relation to the Number of Feedings

**Figure 3:**
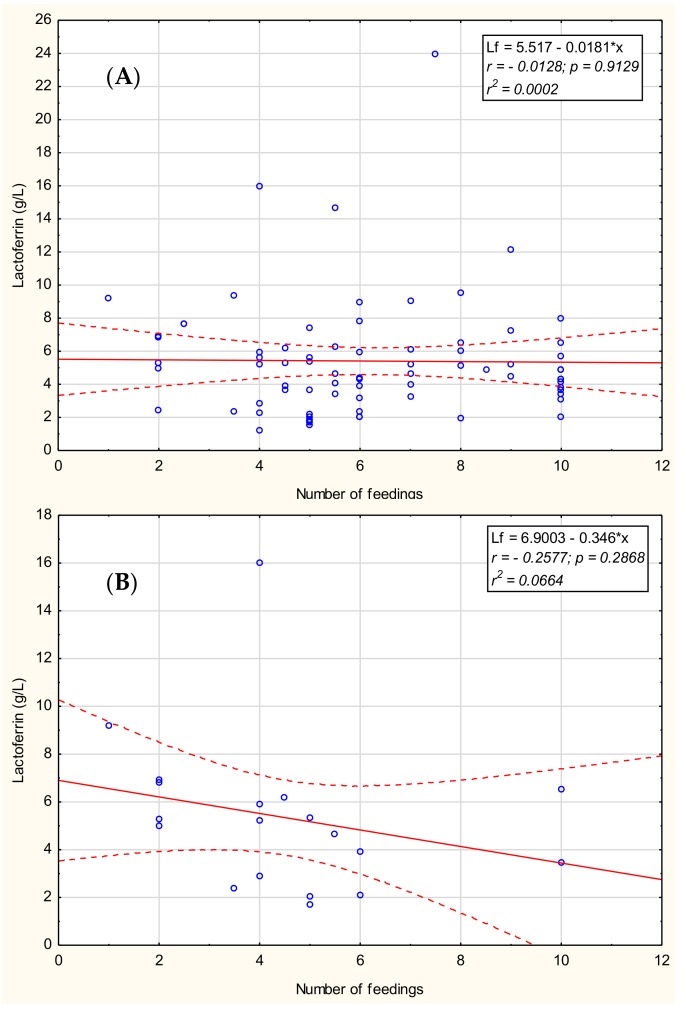
Relationship between the concentration of lactoferrin and the number of feedings mother’s milk (**A**) during 13–48 months of lactation, (**B**) over 24 months of lactation. A solid line indicates linear regression, and 95% confidence intervals are shown by dotted lines; blue hollow—individual samples. *: multiplied.

The relationship between lactoferrin concentration of mother’s milk and the number of feedings during the analyzed period from month 13 to 48 and the three stages of prolonged lactation, namely months 13–18, 19–24, and >24 of lactation were analyzed. We found no significant correlations for months 13–48 (Figure 3A) as well as for months 13–18 and 19–24; however, for lactation over 24 months, we observed a weak negative tendency between the number of feedings and lactoferrin concentration (*r* = −0.26; *p* = 0.2868; Figure 3B). Additionally, we found no correlation between lactoferrin concentration and maternal age, parity, or time of delivery-gestational age.

## 4. Discussion

HM is a dynamic fluid that changes significantly in its composition from the onset of lactation to prolonged lactation above one year postpartum [29,32]. Total milk protein concentration decreases during the lactation period, but this trend is not general for all proteins. In our previous work, we demonstrated that macronutrients concentrations change in prolonged lactation. As a continuation of the previous study, lactoferrin changes in prolonged lactation were assessed in this study. During the first month of lactation, Lf decreases and then remains relatively stable [33]. The highest content of Lf is in colostrum at 5.5 g/L, whereas mature milk contains only 1.5–3.0 g/L, dependent on the stage of lactation in the first year postpartum [32,34,35]. Affolter et al. [36] reported that the concentration of lactoferrin decreases over the lactation period from the fifth day to eight months from 3.30 to 1.17 g/L. However, this decrease was constant during lactation until one to two months (1.24 g/L), and then stabilized until the eighth month. Changes in Lf content may reflect various biological functions of milk during different stages of newborn and infant development [33].

Our results show that the Lf concentration during prolonged lactation ranges from 4.9 to 5.02 g/L. Previous studies have shown that Lf concentration is negatively correlated with the volume of milk expression as well as the stage of lactation and parity [33,37]. The results showed the highest Lf content was recorded between 12 and 24 months of lactation. Above 24 months, concentration decreases, although not significantly, to 4.9 g/L. To the best of our knowledge, this is the first study assessing Lf concentration in human milk during prolonged lactation. These data have shown that Lf content above 12 months of lactation is higher and close to the Lf concentration in colostrum.

The concentration of lactoferrin in mother’s milk showed a positive correlation with protein concentration over lactation from the early stage until four years postpartum. The coefficient of Lf concentration to protein did not show a correlation with lactation progression. Lf concentration and milk compositions are influenced by various factors such as health conditions and/or biochemical indicators of blood [33,34,35,36].

A notable inter-individual variability of lactoferrin content has also been observed, suggesting a lack of tight regulation in the synthesis and/or blood-to-mammary gland transfer of this protein [34]. Thereby, milk enhances the survival of offspring by promoting immunological competence. The rising concentrations of bioactive proteins, which occur as lactation progresses, are likely a physiological response to the diverse environmental demands of the human baby, which progressively acquires nutritional independence [38].

Raw mother’s milk contains a relatively high concentration of Lf, which leads to more effective inhibition of growth of pathogenic bacteria in comparison to pasteurized mother’s milk. As shown by Woodman et al. [38], human Lf is much more effective than bovine Lf in the inhibition of bacterial growth, although higher doses of bovine Lf also showed activity against *Bifidobacterium breve* and *Staphylococcus epidermidis*. Given the above, we suggest that human milk from prolonged lactation, which contains a comparable level of Lf, might be considered an alternative source of milk/lactoferrin provided in the feeding of preterm infants, although further detailed analysis is needed. Different methods used to preserve human milk bioactivity significantly impact Lf concentration [31]. Only further studies involving detailed tests of lactoferrin concentration in milk samples from prolonged lactation before and after pasteurization will help answer this question.

The strength of our study is that it is a unique and well-characterized collection of milk samples of mothers with prolonged lactation of up to 48 months, which impacts the credibility of the results obtained. To determine Lf concentration, a specific immunological method, ELISA, was used to prevent obtaining of false positive results. A limitation of our study is the homogeneous test group, which may not enable drawing a causal relationship between the factors that can influence milk Lf concentration, since, as reported by Yang et al. [33], milk Lf concentrations varies amongst different geographical regions in the Chinese population.

Evidence of stable or increasing immunoprotein levels during prolonged lactation provides the additional argument for allowing non-weaning; however, breastfeeding must be combined with solid foods to meet all the new requirements of a rapidly growing six-month or older baby. A number of clinical trials have been conducted to determine the influence of Lf on the protection against neonatal infections [39,40]. Based on published studies of Lf supplementation in preventing infection, determining the Lf concentration during lactation is important not only in the first six months. If the concentration changes during the lactation phase, colostrum has the highest Lf concentration, and decreases significantly during days after delivery. We now know that the milk of long-nursing mothers has a similar concentration to that in colostrum. Physicians should consider this to encourage exclusive breastfeeding, especially in risk groups such as premature babies [41].

To the best of our knowledge, based on the Internet databases such as Web of Science and pubmed, this is the first large-scale study performed on lactoferrin concentration in breast milk during prolonged lactation and the influence of various factors on its concentration. Coincident with the lack of public acceptance of breastfeeding beyond the first or even the second year, these findings have been ignored for decades.

## 5. Conclusions

Results show that the Lf concentration during prolonged lactation ranges from 4.9 to 5.02 g/L. The highest Lf content was recorded between 12 and 24 months of lactation. Above 24 months, concentration decreases, although not significantly. These data have shown that Lf content above 12 months of lactation is close to the Lf concentration in colostrum.

## Figures and Tables

**Table 1:** Characteristics of the study population.

Outcome and Exposure Variables Breastfeeding	Breastfeeding ≤12 Months *n* = 24% (*n/N*)	Breastfeeding >12 Months *n* = 96% (*n/N*)	χ^2^	*p*-Value	
Maternal age % (*n/N*)					
25–29	50 (12/24)	33 (31/94)	2.672	0.139	NS
30–34	41.7 (10/24)	45 (41/94)			
35+	8.3 (2/24)	22 (22/94)			
Race/ethnicity					
Caucasian	100 (24/24)	100 (96/96)	-	-	-
Socioeconomic status and education					
Secondary education	12.5 (3/24)	20 (16/96)	0.25	0.617	NS
High education	87.5 (21/24)	83.3 (80/96)			
Parity					
1	79 (19/24)	67.7 (65/96)	1.628	0.22	NS
2	21 (5/24)	33.3 (32/96)			
3	0 (0/24)	2 (2/96)			
Birth weight					
Appropriate for Gestational Age (AGA)	100 (24/24)	100 (96/96)	-	-	-
gestational age					
34–37	4.2(1/24)	1. (1/96)	1.144	0.285	NS
>37	95.8(23/24)	99 (95/96)			
Medicines during lactation					
No medications	71(17/24)	66 (63/96)	0.884	0.643	NS
Thyroxine	21(5/24)	29 (28/96)			
Others	8(2/24)	5 (5/96)			
Maternal diet during lactation					
Vegan/vegetarian	0 (0/24)	8 (8/96)	-	-	-
Dairy-free diet	21 (5/24)	7 (7/96)			
Gluten-free diet	0 (0/24)	3 (3/96)			
Complementary foods introduction					
above 6 months of life	NA	94 (90/96)	-	-	-

χ^2^ test at *p* < 0.05; NA—Not assessed, NS—Not significant. -: not calculated

**Table 2:** Lactoferrin and protein concentrations in breast milk during prolonged lactation.

Breast Milk. Lactoferrin/Protein Content	Lactation			
	1–12 Months *n* = 24	13–18 Months *n* = 33	19–24 Months *n* = 37	>24 Months *n* = 26
Lactoferrin (g/L)	3.39 ± 1.43 3.24 2.30–4.47	5.55 ± 4.00 * 4.92 3.69–6.08 *p* < 0.006	5.02 ± 2.97 4.42 3.16–6.53	4.90 ± 3.18 4.29 2.49–6.18
Protein (g/L)	10.5 ± 2.3 10.0 8.5–12.8	10.4 ± 3.4 9.0 8.0–11.0	11.2 ± 2.7 ** 11.0 9.5–12.0 *p* < 0.05	19.1 ± 10.7 *** 14.3 12.0–25.0 *p* < 0.0001
Lactoferrin/Protein ratio	0.32 ± 0.12 0.33 0.25–0.41	0.49 ± 0.21 * 0.54 0.36–0.65 *p* < 0.002	0.43 ± 0.21 0.45 0.22–0.54	0.29 ± 0.17 *** 0.24 0.18–0.36 *p* < 0.009

Values are given as the mean ± SD, median and 25th–75th percentiles. The Mann–Whitney U-test was used for statistical calculations, and a *p*-value lower than 0.05 was considered significant. Significantly different from the milk group of: * 1–12 months of lactation, ** 13–18 months of lactation, *** 19–24 months of lactation.

**Table 3:** Correlations between lactoferrin and macronutrients over prolonged lactation from month 1 to 48 and for particular periods.

Correlation Coefficient (*r* Value *)					
Lactoferrin					
Duration of lactation	1–48 months	1–12 months	13–18 months	19–24 months	Over 24 months
	NS	NS	NS	NS	0.39
Carbohydrate	–0.32	NS	NS	NS	–0.50
Fat	0.19	NS	NS	NS	0.58
Protein	0.25	NS	NS	NS	0.56
True protein	0.24	NS	NS	NS	0.58
Dry mass	0.18	NS	NS	NS	0.65
Energy	0.19	NS	NS	NS	0.65

The values of *r* were calculated according to Spearman’s method corresponding to the correlation between the concentrations of lactoferrin and macronutrients over prolonged lactation from month 1 to 48. * all *r* values are statistically significant with *p* < 0.05. NS—Not significant.

## References

[1] Ballard O., Morrow A.L. (2013). Human milk composition: Nutrients and bioactive factors. Pediatr. Clin. N. Am..

[2] Sorensen M., Sorensen S.P.L. (1939). The proteins in whey. Compte Rendu Trav. Lab. Carlsberg.

[3] Groves M.L. (1960). The isolation of a red protein from milk. J. Am. Chem. Soc..

[4] Johansson B. (1960). Isolation of an iron-containing red protein from human milk. Acta Chem. Scand..

[5] Montreuil J., Tonnelat J., Mullet S. (1960). Preparation and properties of lactosiderophilin (lactotransferrin) of human milk. Biochim. Biophys. Acta.

[6] Rosa L., Cutone A., Lepanto M.S., Paesano R., Valenti P. (2017). Lactoferrin: A Natural Glycoprotein Involved in Iron and Inflammatory Homeostasis. Int. J. Mol. Sci..

[7] Liao Y., Alvarado R., Phinney B., Lönnerdal B. (2011). Proteomic characterization of human milk whey proteins during a twelve-month lactation period. J. Proteome Res..

[8] Lönnerdal B., Iyer S. (1995). Lactoferrin: Molecular structure and biological function. Annu. Rev. Nutr..

[9] Vogel H.J. (2012). Lactoferrin, a bird’s eye view. Biochem. Cell Biol..

[10] Lönnerdal B. (2009). Nutritional roles of lactoferrin. Curr. Opin. Clin. Nutr. Metab. Care.

[11] Zavaleta N., Figueroa D., Rivera J., Julia S., Segundo A., Bo L. (2007). Efficacy of rice-based oral rehydration solution containing recombinant human lactoferrin and lysozyme in Peruvian children with acute diarrhea. J. Pediatr. Gastroenterol. Nutr..

[12] Ochoa T.J., Chea-Woo E., Baiocchi N., Pecho I., Campos M., Prada A., Valdiviezo G., Lluque A., Lai D., Cleary T.G. (2013). Randomized double-blind controlled trial of bovine lactoferrin for prevention of diarrhea in children. J. Pediatr..

[13] Manzoni P., Rinaldi M., Cattani S., Pugni L., Romeo M.G., Messner H., Stolfi I., Decembrino L., Laforgia N., Vagnarelli F. (2009). Bovine lactoferrin supplementation for prevention of late-onset sepsis in very low-birth-weight neonates: A randomized trial. JAMA.

[14] Manzoni P., Garcia Sanchez R., Meyer M., Stolfi I., Pugni L., Messner H., Cattani S., Betta P.M., Memo L., Decembrino L. (2018). Exposure to gastric acid inhibitors increases the risk of infection in preterm very low birth weight infants but concomitant administration of lactoferrin counteracts this effect. J. Pediatr..

[15] Pammi M., Suresh G. (2017). Enteral lactoferrin supplementation for prevention of sepsis and necrotizing enterocolitis in preterm infants. Cochrane Database Syst. Rev..

[16] Mastromarino P., Capobianco D., Campagna G., Laforgia N., Drimaco P., Dileone A., Baldassarre M.E. (2014). Correlation between lactoferrin and beneficial microbiota in breast milk and infant’s feces. Biometals.

[17] Broadhurst M., Beddis K., Black J., Henderson H., Nair A., Wheeler T. (2015). Effect of gestation length on the levels of five innate defence proteins in human milk. Early Hum. Dev..

[18] Villavicencio A., Rueda M.S., Turin C.G., Ochoa T.J. (2016). Factors affecting lactoferrin concentration in human milk: How much do we know?. Biochem. Cell Biol..

[19] Ronayne de Ferrer P.A., Baroni A., Sambucetti M.E., López N.E., Cernadas J.M.C. (2000). Lactoferrin levels in term and preterm milk. J. Am. Coll. Nutr..

[20] Lönnerdal B. (2014). Law infant nutrition: Bioactive proteins of human milk and implications for composition of infant formulas. Am. J. Clin. Nutr..

[21] Law B.A., Reiter B. (1977). The isolation and bacteriostatic properties of lactoferrin from bovine milk whey. J. Dairy Res..

[22] Ostan N.K., Yu R.H., Ng D., Lai C.C.-L., Pogoutse A.K., Sarpe V., Hepburn M., Sheff J., Raval S., Schriemer D.C. (2017). Lactoferrin binding protein B–a bi-functional bacterial receptor protein. PLoS Pathog..

[23] Barbiroli A., Bonomi F., Capretti G.I., Iametti S., Manzoni M., Piergiovanni L., Rollini M. (2012). Antimicrobial activity of lysozyme and lactoferrin incorporated in cellulose-based food packaging. Food Control.

[24] Zarember K.A., Sugui J.A., Chang Y.C., Kwon-Chung K.J., Gallin J.I. (2007). Human polymorphonuclear leukocytes inhibit Aspergillus fumigatus conidial growth by lactoferrin-mediated iron depletion. J. Immunol..

[25] Legrand D. (2016). Overview of Lactoferrin as a Natural Immune Modulator. J. Pediatr..

[26] Wang B., Timilsena Y.P., Blanch E., Adhikari B. (2019). Lactoferrin: Structure, Function, Denaturation and Digestion. Crit. Rev. Food Sci. Nutr..

[27] Hassiotou F., Geddes D.T. (2015). Immune Cell–Mediated Protection of the Mammary Gland and the Infant during Breastfeeding. Adv Nutr..

[28] Vorbach C., Capecchi M.R., Penninger J.M. (2006). Evolution of the mammary gland from the innate immune system?. Bioessays.

[29] Czosnykowska-Łukacka M., Królak-Olejnik B., Orczyk-Pawiłowicz M. (2018). Breast Milk Macronutrient Components in Prolonged Lactation. Nutrients.

[30] Orczyk-Pawiłowicz M., Hirnle L., Berghausen-Mazur M., Kątnik-Prastowska I.M. (2014). Lactation stage-related expression of sialylated and fucosylated glycotopes of human milk α-1-acid glycoprotein. Breastfeed Med..

[31] Wesolowska A., Sinkiewicz-Darol E., Barbarska O., Strom K., Rutkowska M., Karzel K., Rosiak E., Oledzka G., Orczyk-Pawiłowicz M., Rzoska S. (2018). New Achievements in High-Pressure Processing to Preserve Human Milk Bioactivity. Front. Pediatr..

[32] Rai D., Adelman A.S., Zhuang W., Rai G.P., Boettcher J., Lönnerdal B. (2014). Longitudinal changes in lactoferrin concentrations in human milk: A global systematic review. Crit. Rev. Food Sci. Nutr..

[33] Yang Z., Jiang R., Chen Q., Wang J., Duan Y., Pang X., Jiang S., Bi Y., Zhang H., Lönnerdal B. (2018). Concentration of Lactoferrin in Human Milk and Its Variation during Lactation in Different Chinese Populations. Nutrients.

[34] Haschke F., Haiden N., Thakkar S.K. (2016). Nutritive and bioactive proteins in breastmilk. Ann. Nutr. Metab..

[35] Garcia-Rodenas C.L., De Castro C.A., Jenni R., Thakkar S.K., Beauport L., Tolsa J.-F., Fischer-Fumeaux C.J., Affolter M. (2019). Temporal changes of major protein concentrations in preterm and term human milk. A prospective cohort study. Clin. Nutr..

[36] Affolter M., Garcia-Rodenas C., Vinyes-Pares G., Jenni R., Roggero I., Avanti-Nigro O., de Castro C.A., Zhao A., Zhang Y., Wang P. (2016). Temporal changes of protein composition in breast milk of Chinese Urban mothers and impact of caesarean section delivery. Nutrients.

[37] Trend S., Strunk T., Lloyd M., Kok C.H., Metcalfe J., Geddes D.T., Lai C.T., Richmond P., Doherty D.A., Simmer K. (2016). Levels of innate immune factors in preterm and term mothers’ breast milk during the 1st month postpartum. Br. J. Nutr..

[38] Woodman T., Strunk T., Patole S., Hartmann B., Simmer K., Currie A. (2018). Effects of lactoferrin on neonatal pathogens and Bifidobacterium breve in human breast milk. PLoS ONE.

[39] Ochoa T.J., Pezo A., Cruz K., Chea-Woo E., Cleary T.G. (2012). Clinical studies of lactoferrin in children. Biochem. Cell Biol..

[40] Siqueiros-Cendón T., Arévalo-Gallegos S., Iglesias-Figueroa B.F., García-Montoya I.A., Salazar-Martínez J., Rascón-Cruz Q. (2014). Immunomodulatory effects of lactoferrin. Acta Pharmacol. Sin..

[41] Newburg S., Walker W.A. (2007). Protection of the neonate by the innate immune system of developing gut and of human milk. Pediatr. Res..

